# Special Issue: Advances in Opportunistic Viral Infections II

**DOI:** 10.3390/v17060739

**Published:** 2025-05-22

**Authors:** Carlo Contini, Roberto De Giorgio, Matteo Guarino, Martina Maritati

**Affiliations:** 1 Infectious Diseases, Department of Medical Sciences, University of Ferrara, 44124 Ferrara, Italy; 2 Department of Translational Medicine, University of Ferrara, 44124 Ferrara, Italy; roberto.degiorgio@unife.it (R.D.G.); matteo.guarino@unife.it (M.G.); martina.maritati@unife.it (M.M.)

Since the 1980s, particular opportunistic viral infections (OVIs) have been associated with HIV/AIDS infection, a pandemic still not eradicated as late diagnoses continue to emerge each year, leading to a high mortality and lethality rate [1]. Today, however, there are OVIs that arise in people who do not have HIV/AIDS but who are vulnerable because they are immunocompromised, have received an organ transplant, or are undergoing prolonged treatment with biotech or chemotherapy drugs.

This Special Issue focuses on some important OVIs which occur in specific settings such as immunocompromised adult and pediatric subjects, including frail individuals and organ transplant recipients. It features eight papers providing insights into the epidemiology, pathogenesis and treatment of SARS-CoV-2, hepatitis B virus (HBV) infection, human polyoma virus 1 (BK), and herpesviruses in specific settings.

Coudray MS et al. [2] studied the effects of vitamin D on the COVID-19 burden and the possibility of supplementing the general population with vitamin D to reduce the impact of COVID-19. Recently, in fact, the link between vitamin D and the outcome of COVID-19 has attracted a fair amount of attention. Indeed, published studies have demonstrated a clinically significant association between vitamin D deficiency and increased severity of SARS-CoV-2 infection [3], which is not found when vitamin D levels are adequate. In this regard, the authors explored the relationship between vitamin D levels and SARS-CoV-2 infection in the Minority and Rural Coronavirus Insights Study (MRCIS) cohort, a heterogeneous population of medically underserved people who presented at five federally qualified health centers in the United States. It was found that lower vitamin D consumption was significantly associated with more severe symptoms of SARS-CoV-2 infection, whereas, when average vitamin D levels were increased, the majority of enrolled subjects with SARS-CoV-2 infection had less severe clinical manifestations. Other authors found that in a large population of people with medical deficiencies, for every 10-unit increase in vitamin D levels and taking into account race/ethnicity and age, the odds of SARS-CoV-2 infection decreased by 12 percent, thus agreeing with the existing literature that has shown that vitamin D deficiency increased the risk of infection with this coronavirus [4]. Moreover, Hispanic/Latino and non-Hispanic black participants had a higher likelihood of infection compared with non-Hispanic whites after adjustment for race/ethnicity and age.

A very original and interesting aspect concerns the epidemiological and clinical profile of COVID-19 patients admitted during the three waves of the pandemic in a tertiary care center in Amazon regions of Brazil [5]. This descriptive, observational, and cross-sectional study aimed to compare the most relevant clinical and epidemiological profiles of individuals affected by SARS-CoV-2 infection and hospitalized in a public hospital in the city of Belém, Pará, in Amazon regions of Brazil over the course of the various waves. The presence of comorbidities (hypertension and diabetes), advanced age, and male sex (particularly those over 40 years of age) were important factors in the severity and need for hospitalization of these patients (both the pulmonary and extrapulmonary symptoms were more frequent), despite a gradual reduction in later waves. This study also corroborated that the implementation of the vaccination policy was a key factor in reducing the number of hospitalizations [6].

Another important aspect related to COVID-19 concerns the impact and evolution of infection during the various pandemic waves in liver-transplant-recipient patients, who are well known to be chronically immunosuppressed patients with frequent associated comorbidities [7]. Through an observational study conducted on a large cohort of patients with COVID-19 admitted to a university hospital in Madrid, Spain, in order to assess clinical changes and risk factors during the different waves of the COVID-19 pandemic in the three-year period from 2020 to 2023, the authors found that among various comorbidities considered independently, including renal disease with creatinine ≥ 2 mg/dL, COPD, type 2 diabetes mellitus, and history of cardiovascular events, older age and creatinine values > 2 mg/dL represented the risk factors most responsible for respiratory failure (also worsened by taking mycophenolate mofetil and calcineurin inhibitors) and hospitalization. As previously shown, this study also pointed out that vaccination was a protective factor for hospitalization, respiratory failure and mortality, the latter peaking at 21.4 percent during the fourth pandemic wave [8,9].

The other five manuscripts deal with Epstein–Barr virus (EBV), hepatitis (HBV), human polyoma virus 1 (BKV), and herpesviruses infections in particular adult and pediatric clinical settings, including lactating women.

The first of these [10] aimed to evaluate current treatment modalities and evolving strategies for the use of EBV as a biomarker for the screening and prevention of post-transplant lymphoproliferative disorder (PTLD), one of the most common malignancies in pediatric liver transplant recipients, associated with significant morbidity and mortality, as is often concomitant with EBV, which strongly increases the risk of developing PTLD [11]. The authors explored and highlighted evolving strategies for the use of EBV-DNAemia (NAAT-PCR) as a biomarker of PTLD or other potential biomarkers (EBER flow fluorescence in situ hybridization (EBER flow FISH)) for the screening and prevention of PTLD in children found to be at increased risk for this disorder.

On the topic of organ transplantation, Myers et al. [12] aimed to demonstrate the feasibility of an updated model of human polyomavirus 1 infection, known as BK virus (BKPyV), in kidney transplant recipients requiring a lifelong immunosuppression protocol to prevent rejection, using all recently available literature data. The researchers described the datasets provided by the Duke Transplant Center and the National Institute of Health’s Clinical Trials in Organ Transplantation and the subset of patient data used to calibrate the new model [13]. The results of this paper demonstrated improvements to an existing immune response model for BKPyV-sensitive renal transplant recipients, reducing its complexity and retaining the ability to capture BKPyV dynamics and serum creatinine levels found in the data, thus providing clinicians with a tool that can not only predict outcomes of different treatment strategies but also help prescribe optimized immunosuppression treatments for individual patients.

Another aspect poorly investigated, but which may soon be the subject of in-depth studies, concerns the high prevalence of HBV resistance-associated mutations (RAMs) to lamivudine in HIV/HBV-coinfected people in rural and peri-urban communities in Botswana, an African region that has an HIV prevalence of 20.8 percent in the adult population [14]. The authors identified HBV RAMs in 61/98 (62.2%) of the enrolled subjects. Most RAMs were in positions 204 (60.3%), 180 (50.5%) and 173 (33.3%), mostly associated with lamivudine resistance. Hence, the high prevalence of RAMs to lamivudine discourages the use of ART regimens with 3TC as the only active HBV drug in HIV-co-infected persons, particularly those known to confer resistance to 3TC. Consequently, people displaying a continuously high HBV viral load should be tested for RAMs to HBV drugs.

The authors from University Estadual de Campinas (UNICAMP, Brazil) studied and estimated the occurrence, patterns, and outcomes of EBV, HCMV, human herpesvirus 6 (HHV-6 type A and type B) and 7 (HHV-7) polyomavirus (BKV), and human adenovirus (HAdV) in children with nephrotic syndrome (NS) characterized by severe proteinuria, hypoalbuminemia, hyperlipidemia, and edema, as the significance of OVIs in these clinical settings is yet to be defined [15]. This interesting study found that active HCMV infection was the most frequent (54.5%), while other herpesviruses such as HHV-6, BKV, HHV-7, EBV and HAdV had a lower frequency (27.3%, for HHV-7, 18.2% for EBV and HAdv and 9% for HHV-6, respectively). Therefore, some OVIs, particularly HCMV, may be an important cause of morbidity and relapse of NS in children, which, in most cases, lead to hospitalization, with increased likelihood of mortality [16].

An original review based on an extensive literature search conducted on PubMed, Cochrane Library, and Scopus evaluated the safety and efficacy of antiviral therapies used to manage Herpesviridae infections, including HSV, VZV, CMV, and EBV, in nursing mothers [17]. Assessing the current evidence in the literature, the authors sought to identify best practices and research gaps and ultimately guide future studies to ensure safer and more effective antiviral treatment options for both mothers and their infants [18]. This study suggested that antiviral agents, particularly acyclovir and valacyclovir, are generally safe for lactating mothers, with minimal infant exposure and low risk of adverse effects. However, EBV infections in immunocompetent individuals are typically self-limited and rarely require pharmacological treatment, even if lactation has been identified as a potential transmission route for EBV.

Further studies will be needed to provide accurate data on the actual clinical effectiveness of antiviral treatments.
